# Molecular analysis of ovarian mucinous carcinoma reveals different cell of origins

**DOI:** 10.18632/oncotarget.5146

**Published:** 2015-09-02

**Authors:** Yihong Wang, Lauren Ende Shwartz, Derek Anderson, Ming-Tseh Lin, Lisa Haley, Ren-chin Wu, Russell Vang, Ie-ming Shih, Robert J. Kurman

**Affiliations:** ^1^ Department of Pathology, Sir Run Run Shaw Hospital, Zhejiang University School of Medicine, Hangzhou, 310016, China; ^2^ Department of Pathology, Johns Hopkins Medical Institutions, Baltimore, 21231, MD, USA; ^3^ Department of Pathology, Chang Gung Memorial Hospital and Chang Gung University College of Medicine, Taoyuan, 33378, Taiwan; ^4^ Department of Gynecology and Obstetrics, Johns Hopkins Medical Institutions, Baltimore, 21231, MD, USA; ^5^ Department of Oncology, Johns Hopkins Medical Institutions, Baltimore, 21231, MD, USA

**Keywords:** ovarian, mucinous carcinoma, teratoma, microsatellite genotyping, HUMARA assay

## Abstract

It is believed that a subset of primary ovarian mucinous tumors is derived from mature teratomas [1–5]. To confirm this, we performed microsatellite genotyping using a variety of short tandem repeat makers and analyzed allelotypes of 8 mucinous tumors (4 mucinous carcinomas, 3 atypical proliferative mucinous tumors and 1 mucinous cystadenoma) associated with a teratoma to determine whether they were clonally related. 7 of the 8 mucinous tumors showed complete or a high degree of homozygosity. Among the 6 pairs of tumors with teratoma tissue available for comparison, 5 of 6 showed a high or complete degree of allelotypes matching, which differed from the somatic allelotypes of the normal control tissue. A discrepancy was detected between carcinoma and teratoma in one pair at several loci, with different X-chromosome inactivation patterns revealed by the HUMARA clonality assay. We also investigated the allelotypes of 16 ovarian mucinous carcinomas without a teratoma in young patients (range 13–30) and in 6 older patients (range 40–67) using the same method. None of these tumors showed pure homozygosity. The number of homozygous loci in this cohort was significantly lower than that in the first. Our results suggest first, that most mucinous tumors associated with a teratoma are derived from the teratoma but occasionally they could be collision tumors and second that the majority of pure mucinous tumors in young women in whom a teratoma is not present are not derived from a teratoma.

## INTRODUCTION

Ovarian mucinous tumors, including cystadenoma, atypical proliferative (borderline) mucinous tumor (APMT) and mucinous carcinoma, unlike other ovarian epithelial tumors, frequently occur in young women [3, 6]. In addition, a subset of mucinous tumors is associated with teratomas and immunohistochemical studies showing that they have similar immunoprofiles supporting the interpretation that the mucinous tumors develop from mucinous epithelium in the teratoma [1–4]. Although these mucinous neoplasms are generally regarded as primary in the ovary the possibility of a metastasis from a colorectal carcinoma cannot be entirely excluded because their immunophenotypes can be identical [1–4]. To prove that this subset of mucinous tumors is derived from the associated teratoma, we performed microsatellite genotyping in 8 cases of combined mucinous tumors and teratomas and compared the allelotypes of each tumor to its normal control tissue at each short tandem repeats (STR) loci to determine whether or not the tumors were clonally-related.

We also speculated that mucinous tumors in young women in whom a teratoma was not detected may have arisen from a teratoma in which the teratomatous component was overgrown and obliterated by the mucinous neoplasm. However, little is known about zygosity of this entity, with the exception of one recent study investigating a cohort of patients ranging from 31–76 years, mean age 54.4[7], which showed heterozygosity in all the tumors. To test this hypothesis, we investigated the allelotypes of 16 ovarian mucinous carcinomas in young patients (13–30 years) and in 6 older patients (40–67 years) using the same method discussed above.

## RESULTS

**Figure 1:**
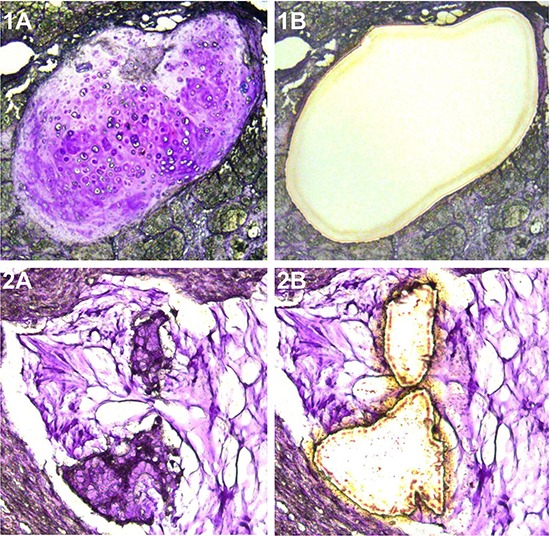
Microdissected teratoma and mucinous carcinoma component analyzed **A.** Cartilagenous tissue dissected from mature cystic teratoma. **B.** Ruptured mucinous carcinomatous gland embedded in acellular mucin pool observed in a mucinous carcinoma associated with a teratoma.

A cohort of 8 ovarian mucinous tumors (4 mucinous carcinomas, 3 APMTs and 1 mucinous cystadenoma) associated with mature cystic teratomas were included in this study (Table 1, Figure 1). The mean patient age was 35 years (range 16–57). Except for one mucinous carcinoma that was bilateral all the other tumors were unilateral. The two tumor components were of admixed on gross examination in 4 of the 8 cases while in the remaining 4 cases the tumors were close to one another but distinctly separate. In 3 cases bland mucinous epithelium was present in the teratoma (case 2, 4 and 6). Five of the 8 mucinous tumors (3 carcinomas and 2 APMT) in this cohort displayed pseudomyxoma ovarii. Another cohort of 22 ovarian mucinous carcinomas without a teratomatous component was also analyzed (Table 2, mean age 30.6; range 13–67), among which 16 patients were 30-years-old or younger (range 13–30). None of the tumors in this group were associated with pseudomyxoma ovarii. All the mucinous tumors in this study displayed an intestinal phenotype and there was no evidence of extra-ovarian disease.

**Table 1:** Clinical and pathological features of mucinous tumors associated with mature cystic teratoma

Case	Age	Location	Histology of Mucinous Tumor /Mucinous Component in Teratoma?	Spatial Relationship of the Tumors	Tumor Grade	MC/APMT in Mucinous Tumor?	Pseudomyoma Ovarii?
1	16	RO	MC/N	Admixed	N/A	N/A	N
2	57	RO	APMT/Y	Admixed	N/A	N/A	Y
3	57	LO	APMT/N	Adjacent	N/A	N/A	Y
4	30	RO	APMT/Y	Admixed	N/A	N/A	N
5	32	LO	Carcinoma/N	Adjacent	1	Y	Y
6	28	LO	Carcinoma/Y	Admixed	1	Y	Y
7	46	RO	Carcinoma/N	Adjacent	2	Y	Y
8	23	BO	Carcinoma//N	Adjacent	2	Y	N

LO: Left ovary; RO: Right ovary; BO: Bilateral ovary; MC: Mucinous cystadenoma; APMT: Atypical proliferative mucinous tumor; Y: Yes; N: No; N/A: Not applicable.

**Table 2:** Clinical and pathological features of mucinous carcinoma unassociated with teratoma

Case	Age	Site	Tumor Grade	MC/APMT present?	Pseudomyxoma ovarii?
YP					
1	22	LO	1	Y	N
2	22	LO	1	Y	N
3	27	LO	1	N	N
4	29	LO	1	Y	N
5	27	LO	3	Y	N
6	30	LO	2	Y	N
7	16	LO	1	Y	N
8	18	RO	1	Y	N
9	24	LO	1	Y	N
10	30	RO	1	Y	N
11	29	LO	2	Y	N
12	13	LO	2	N	N
13	30	LO	1	Y	N
14	15	RO	2	Y	N
15	28	RO	2	N	N
16	27	LO	2	N	N
OP					
17	58	LO	1	Y	N
18	47	LO	1	N	N
19	50	RO	1	Y	N
20	54	RO	1	N	N
21	40	LO	1	Y	N
22	67	LO	2	Y	N

MC: Mucinous cystadenoma; APMT: Atypical proliferative mucinous tumor; YP: Young patients; OP: Older patients; LO: Left ovary; RO: Right ovary; Y: Yes; N: No.

**Figure 2:**
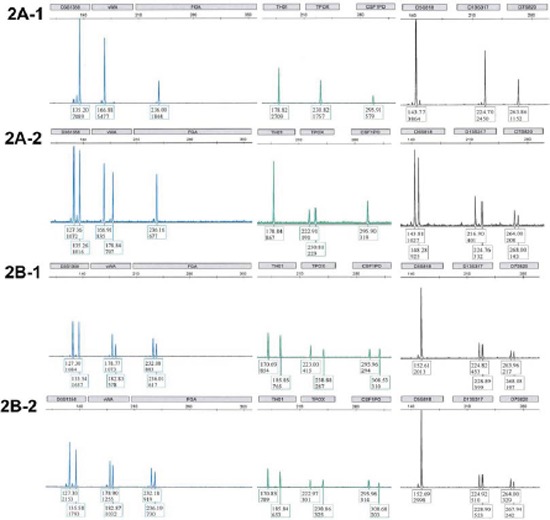
Allelotypes of representative cases of mucinous carcinomas at 9 STR loci are shown in capillary electropherogram The locus with a heterozygous pattern in the control tissue was considered informative. Upper panel: Mucinous carcinoma **2A-1.** associated with teratoma and normal fallopian tube **2A-2.** The carcinoma sample displayed homozygosity at all 6 informative loci (D3S1358, vWA, TPOX, D5S818, D13S317 and D7S820) while the rest 3 loci were homozygous in control (fallopian tube) tissue and considered uninformative. Lower panel: Mucinous carcinoma **2B-1.** unassociated with teratomas and normal fallopian tube **2B-2.** The carcinoma sample showed heterozygosity at all eight informative loci (D3S1358, vWA, FGA, TH01, TPOX, CSF1PO, D13S317 and D7S820).

**Figure 3:**
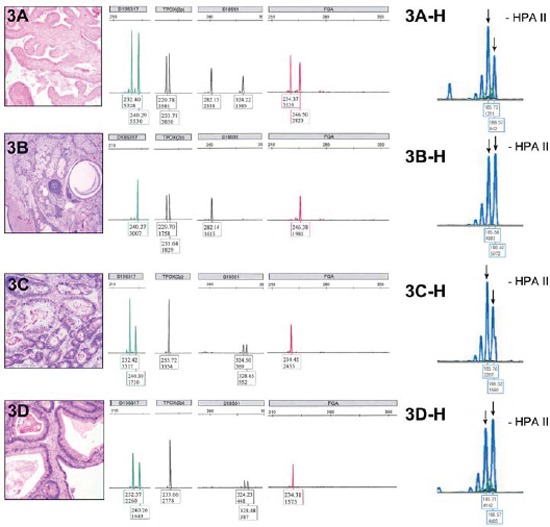
Allelotypes of different tumors at representative STR Loci and HUMARA assay of case 8 Fallopian tube **3A.** teratoma in the right ovary **3B.** mucinous carcinoma from left **3C.** and right ovary **3D.** were dissected to extract DNA. Left panel: capillary electropherogram showed that at the loci of D18S51 and FGA, different single alleles were present between the two tumors (3B, 3C, 3D) while the control tissue sample showed two alleles (3A). The two adjacent small peaks in the two carcinomas from bilateral ovaries denoted a STR “biallelic” artifact, suggesting a possible somatic mutation. The teratoma (3B) was homozygous at D13S317 while carcinoma (3C, 3D) was heterozygous. The teratoma (3B) was heterozygous at TPOX locus while carcinoma (3C, 3D) was the homozygous. The allelotypes of carcinoma samples from both ovaries (3C, 3D) completely matched. HUMARA assay showing different XCI patterns between teratoma and mucinous carcinoma **3A-H, 3B-H, 3C-H.** and **3D-H.** The electropherogram shows the fragment analysis of PCR products amplified from undigested and digested DNA of control tissue (3A-H), teratoma (3B-H) and carcinoma from both sides(3C-H, 3D-H). 2 major peaks represented 2 alleles with different numbers of short tandem repeats at the HUMARA locus (black arrows). After digestion, the control DNA retained a balanced XCI pattern (3A-H), teratoma sample displayed preferential loss of the short allele (3B-H, red arrow) and carcinoma samples showed complete loss of the long alleles(3C-H, 3D-H, red arrows).

Detailed microsatellite genotyping results at 9 tested loci are shown in Table 3. In the cohort of 8 mucinous tumors associated with teratoma there were only 6 in which teratomatous tissue was available for analysis. Tumors 1–5 showed completely matching allelotypes with the corresponding teratomatous components. In this group, tumors 1, 2, 3 and 5 showed complete isodisomy at all informative loci. In tumors 6 and 7, the carcinoma samples also displayed a high degree of isodisomy at the informative loci (100%; 80%) although the teratoma DNA was not available for comparison. None of the 8 mucinous tumors were completely heterozygous at the 9 loci tested. In total (pairs1–7), 92.5% (37/40) of informative loci were homozygous, the mean number of homozygous loci was 5.29 (range 4–6). The capillary electropherogram of two representative cases of mucinous carcinomas were shown in Figure 2. In pair 8, discrepancy at 4 STR loci was identified between the teratoma and carcinoma components (Figure 3). At the locus of D18S51 and FGA, different single alleles were present between the two tumors. The position of the two adjacent small peaks at D18S51 in the carcinoma overlapped with that of the long allele in control tissue, denoting a STR “biallelic” artifact most commonly reported at this locus, suggesting a possible somatic mutation [8]. The teratoma was homozygous at D13S317 while the carcinoma was heterozygous. The teratoma was heterozygous at TPOX locus while carcinoma was homozygous. The allelotypes of the carcinoma samples from both ovaries completely matched. In addition, there were no matching homozygous loci detected between the 2 tumors. Further the HUMARA clonality assay of the mucinous carcinoma and teratoma samples revealed different X-chromosome inactivation (XCI) patterns between the teratoma and carcinomas while the carcinoma involving both ovaries shared the same pattern (Figure 3A–H, Figure 3B–H, Figure 3C–H and Figure 3D–H). The corrected cleavage ratio of the teratoma sample was 3.03, indicating a significant reduction (>70%) of the short allele and predominance of the long allele, while in the carcinoma samples from both ovaries only the short alleles were present.

**Table 3:** Microsatellite genotyping of ovarian mucinous tumor at 9 Microsatellite loci

Case		D3S1358	vWA	FGA	TH01	TPOX	CSF1PO	D5S818	D13S317	D7S820
Pair1	MC	■	■	■	☆	■	■	☆	☆	■
	Teratoma	■	■	■	☆	■	■	☆	☆	■
Pair2	APMT	■	■	■	☆	☆	■	☆	■	☆
	Teratoma	■	■	■	☆	☆	■	☆	■	☆
Pair3	APMT	■	■	■	●	■	☆	●	■	▬
	Teratoa	■	■	■	●	■	☆	●	■	▬
Pair4	APMT	■	■	■	☆	☆	☆	■	■	☆
	Teratoma	■	■	■	☆	☆	☆	■	■	☆
Pair5	Carcinoma	■	■	■	☆	☆	☆	■	■	■
	Teratoma	■	■	▬	☆	☆	☆	■	■	■
Pair6	Carcinoma	■	■	☆	☆	■	☆	■	■	■
	Teratoma	▬	▬	▬	▬	▬	▬	▬	▬	▬
Pair7	Carcinoma	☆	●	■	☆	☆	■	☆	■	■
	Teratoma	▬	▬	▬	▬	▬	▬	▬	▬	▬
Pair8	Carcinoma (l)	●	●	▲	●	■	●	☆	●	●
	Carcinoma (r)	●	●	▲	●	■	●	☆	●	●
	Teratoma (r)	●	●	■	●	●	●	☆	■	●
Carcinoma 1		☆	☆	☆	●	●	☆	●	●	●
Carcinoma 2		●	☆	☆	●	●	●	●	●	●
Carcinoma 3		☆	●	●	●	☆	●	●	●	☆
Carcinoma 4		●	■	●	●	●	●	▬	■	▬
Carcinoma 5		☆	●	☆	●	▬	▬	●	☆	●
Carcinoma 6		●	☆	●	●	☆	☆	☆	■	▬
Carcinoma 7		●	●	●	●	☆	☆	●	●	☆
Carcinoma 8		●	■	●	●	●	☆	☆	●	●
Carcinoma 9		●	●	●	●	●	●	☆	●	●
Carcinoma 10		☆	■	●	■	☆	●	☆	●	■
Carcinoma 11		☆	☆	●	☆	☆	☆	●	●	●
Carcinoma 12		●	●	●	●	●	●	●	●	●
Carcinoma 13		☆	■	■	●	●	●	●	●	●
Carcinoma 14		☆	●	●	●	■	●	●	●	●
Carcinoma 15		☆	●	●	●	☆	■	■	■	●
Carcinoma 16		●	●	☆	☆	●	☆	●	●	▬
Carcinoma 17		■	●	●	■	☆	▬	●	●	●
Carcinoma 18		●	☆	☆	☆	▬	▬	●	●	☆
Carcinoma 19		●	●	☆	●	●	●	●	●	☆
Carcinoma 20		●	☆	☆	●	■	■	☆	●	■
Carcinoma 21		☆	●	●	●	●	●	☆	●	●
Carcinoma 22		●	●	●	●	●	☆	●	●	☆

APMT: Atypical proliferative mucinous tumor; MC: Mucinous cystadenoma; l: left; r: right. Allelotypes of each tumor at 9 tested short tandem repeat loci are indicated as different shapes, with star indicating uninformative loci, square and triangle indicating homozygous loci with different allele present, circle indicating heterozygous loci and hyphen indicating loci failed to PCR amplify. Loci were considered heterozygous when the 2 alleles in the tumor tissue matched 2 alleles in the control tissue. In contrast, loci were considered homozygous when 1 allele was seen in tumor tissue while 2 alleles were seen in control tissue. Uninformative loci represented those with only one allele present in the normal control.

In the cohort of mucinous carcinoma without a teratoma, no purely homozygous carcinoma was detected in either the young (carcinoma 1–16) or older patients (carcinoma 17–22). Ten out of 16 (62.5%) tumors in the group of young women and 3 out of the 6 (50%) in the older group of women were completely heterozygous. In the total of 22 cases of mucinous carcinomas within this cohort, 13% (18/139) of informative loci were homozygous, the mean number of homozygous loci was 0.91 (range 0–3). The number of homozygous loci between young and elder patients in this cohort was not significantly different (*p* = 0.20). However, the number of homozygous loci between cohorts of mucinous tumors unassociated and associated with teratoma was significantly different (*p* < 0.01).

## DISCUSSION

Clinicopathologic and molecular genetic studies have shown that most epithelial ovarian carcinomas develop from precursor lesions that are müllerian derived which is consistent with their müllerian phenotype. Accordingly, serous tumors are thought to develop from precursor lesions in the fallopian tube and it is generally accepted that endometrioid, clear cell and seromucinous tumors arise from endometriosis [9–14]. The pathogenesis of mucinous carcinoma, however, is not well established. It has been proposed that a subset arises from mature teratomas, 50–100% of which showing a CK7-/CK20+ phenotype [1, 5], would mimic metastatic colorectal carcinomas and therefore this can lead to difficulty in diagnosis of a primary versus a metastatic carcinoma. The presence of a teratomatous component is generally interpreted as evidence of ovarian origin, especially in young women with unilateral tumors. On the other hand, as mature teratomas are a common ovarian tumor in these individuals, it is conceivable that some are collision tumors.

In the majority of teratoma-associated mucinous tumors in this study, the complete or high degree of homozygosity and concordance in zygosity with the teratomatous components, is consistent with the findings of Kerr et al. and Fujii et al. [7, 15] and supports their germ-cell origin. However, in case 8, the 23-year-old patient with a unilateral teratoma and bilateral mucinous carcinoma, the discrepancy of allelotypes at loci of D18S51, FGA and D13S317 between the teratoma and carcinoma strongly suggests that the two tumors originated from different cellular clones although the difference at the locus of TPOX could be a result of loss of heterozygosity (LOH). The different XCI patterns revealed by the HUMARA clonality assay also supports the interpretation that the tumors are not genetically related while the concordant XCI pattern between the bilateral tumors is consistent with their shared clonality [16–22]. Although there was no evidence of carcinoma elsewhere, this case could nonetheless represent a metastatic mucinous carcinoma from an occult primary [23–25], especially considering the fact that the tumors were bilateral. Another possibility, although much less likely in the absence of extra-ovarian disease, is that the carcinoma originated from one ovary, and was associated with a teratoma (a collision tumor) and metastasized to the other ovary.

There was no support for our hypothesis that mucinous tumors in young women that were not associated with a teratoma could have arisen from a teratoma that was subsequently obliterated by an expanding mucinous neoplasm. The finding was consistent with Kerr's study [7], which analyzed 9 cases of intestinal type ovarian mucinous carcinoma without teratoma, however only in an older patient group (mean age 55.4, range 31–76 years old). The majority of the tumors in this cohort showed heterozygous allelotypes, which is consistent with the somatic cell allelotype of the normal control tissue. The 1–3 homozygous loci in each case identified in 9 of 22 cases could be explained by LOH, a frequent molecular event in carcinoma. Combining Kerr's study with ours[7], in a total of 31 cases of ovarian mucinous carcinoma, 16 young and 15 older, the carcinomas in the young women did not show significant differences in terms of homozygous loci numbers compared to the older women (*p* = 0.26), although the number of cases is still relatively small. It is therefore likely that these carcinomas developed from a non-germ cell precursor lesion. It should be noted that teratomas can demonstrate various degrees of host isodisomy, depending on their underlying meiotic errors and molecular genetic makers used for testing [7, 15, 26–30]. For example, the teratoma in case 8 displayed only 2 homozygous loci among 8 informative loci included in the AmpFLSTR Prifiler kit. This partial homozygosity could be explained either by contamination of somatic tissue or suppression of meiosis II following meiosis I, leading to the development of a teratoma [26–30]. In fact, a minority of teratomas generated from meiosis I failure could be of identical zygosity of the host (somatic) tissue [28–30]. We favor the latter explanation as pure sebaceous gland tissue, devoid of lymphocytes, were laser capture micro-dissected and used for this case to extract DNA. It is thus conceivable that a carcinoma derived from the teratoma would also display 2 homozygous loci at the most, if there were no LOH. Therefore, the possibility of germ-cell origin of some, if not all, of the tumors in this cohort could not be completely excluded by this method.

In addition, the high prevalence of pseudomyxoma ovarii (71.4%, 5 out of 7) in atypical proliferative mucinous tumors and mucinous carcinomas associated with teratomas in our study (Case 1–7) and complete absence in those not associated with a teratoma suggests that pseudomyxoma ovarii in association with a unilateral mucinous carcinoma or atypical proliferative mucinous tumor may originate from a teratoma. If not detected initially, additional sections can be performed in an effort to detect a teratoma.

In conclusion, our study shows that most mucinous tumors associated with a teratoma are derived from the teratoma but that on occasion they may be collision tumors. Furthermore, ovarian mucinous carcinomas unassociated with teratoma in both young and older women mostly likely are of nongerm cell in origin, however, our numbers were relatively small and therefore study of more cases using different types of molecular analysis is necessary to definitively support this interpretation.

## MATERIALS AND METHODS

### Tissue specimen collection

We retrieved formalin-fixed paraffin-embedded (FFPE) tissue blocks from 8 ovarian mucinous tumors associated with teratoma and 22 cases of ovarian mucinous carcinoma not associated with a teratoma from the consult and in-house surgical pathology archives of The Johns Hopkins Hospital. Slides were reviewed by three gynecologic pathologists (LES, RV and RJK). A total of 34 FFPE blocks was collected from the tumors and 30 FFPE blocks from fallopian tubes, which served as normal controls. The study was approved by the Institutional Review Board of The Johns Hopkins Hospital.

### Laser capture microdissection, macrodissection and DNA extraction

Twelve micron-thick sections were obtained from FFPE tissue blocks of the tumors, placed on membrane slides (Carl Zeiss MicroImaging, Göttingen, Germany) and counterstained with hematoxylin. Tumour cells from mucinous neoplastic epithelium and teratoma were microdissected using the PALM laser capture microdissection microscope (Leica Microsistem, LMD 7000). For normal control tissue, 10 micron-thick sections of fallopian tube tissue were obtained from the FFPE blocks. After 24 h of proteinase K digestion, genomic DNA was extracted using a QIAamp DNA FFPE Kit (Qiagen, Valencia, CA) following the vendor's protocol.

### Microsatellite genotyping analysis

Nine microsatellite loci (D3S1358, vWA, FGA, TH01, TPOX, CSF1PO, D5S818, D13S317 and D7S820) were PCR amplified using AmpFLSTR Prifiler kit (Applied Biosystems, Foster City, CA) in the 8 ovarian mucinous tumors associated with mature teratoma and 22 cases of ovarian mucinous carcinoma. 6 additional loci (D8S1179, D21S11, D7S820, D16S539, D2S1338 and D19S433) were tested in case 8 in this cohort using AmpFLSTR Identifiler kit (Applied Biosystems, Foster City, CA). The following thermocycling conditions were used: initial denaturation at 95°C for 1 minute, followed by 28 cycles at 94 for 1 minute, 59°C for 1 minute, and 72°C for 1 minute, followed by a final extension at 60°C for 45 s. After multiplex PCR amplification, 1 μl of PCR product was mixed with 9 μl of HiDi formamide/GeneScan 500 [ROX] size standard mixture (Applied Biosystems, Foster City, CA, USA). Fragments sizes were analyzed on the ABI3130xl Genetic Analyzer (Applied Biosystems, Foster City, CA, USA). Each locus displaying 2 major peaks were considered heterozygous and homozygous if only 1 major peak was shown. The genotype of each tumor was compared to that of normal control tissue for each of the loci tested. Only the locus with a heterozygous pattern in the control tissue was considered informative. The locus was considered uninformative if the control tissue displayed a homozygous allelotype or if the amplicon was insufficient for a certain allele size call.

### HUMARA ASSAY (DNA digestion and PCR)

HUMARA assay was performed in pair 8 by using the following protocol: 5–10 μl DNA from each tumor and control tissue were incubated for 16 hours at 37°C in a 25 μl reaction containing 10 U of the methylation-sensitive restriction enzyme HpaII (New England Biolabs, Ipswich, MA). In parallel, each sample was subjected to mock digestion in buffer devoid of restriction enzyme. The reaction was terminated by incubation at 80°C for 20 minutes according to the manufacturer's instructions, followed by purification using QIAquick PCR Purification Kit (Qiagen, Valencia, CA). Both digested and mock-digested DNAs were PCR amplified (Applied Biosystems, Foster City, CA) using the following thermocycling conditions: initial denaturation at 94°C for 30s, followed by 40 cycles at 94 for 30 s, 62°C for 45 s, and 68°C for 60 s. All PCRs were finally elongated at 68°C for 5 min. The amplification primers used for HUMARA locus were as follows: 5′-TCCAGAATCTGTTCCAGAGCGTGC-3′ (sense) and 5′-CTCTACGATGGGCTTGGGGAGAAC-3′ (antisense). The antisense primer was labeled with 6FAM at the 5′ end. Capillary electrophoresis of the PCR products was performed on an ABI 3130 XL Genetic Analyzer Sequencer (Applied Biosystems, Foster City, CA) and fragment sizes were analyzed with the use of GeneMapper Software vs 5 (Applied Biosystems, Foster City, CA). The patient was considered heterozygous and informative when the PCR of undigested DNA from the control tissue showed two major peaks, representing the maternal and paternal alleles respectively. To compensate for possible preferential amplification of the shorter allele, we calculated CR_tumor_ (cleavage ratio of tumor) and CR_ctrl_ (cleavage ratio of control tissue) by using the following formula: (peak1 height of undigested sample÷peak2 height of undigested sample) ÷ (peak 1 height of digested sample÷peak 2 height of digested sample) [13–14]. To compensate for possible constitutional XCI skewing, CR_cor_ (corrected cleavage ratio of tumor) was calculated using the following formula: CR_tumor_ ÷ CR_ctrl_ [14]. CR_cor_ of more than 2 or less than 0.5, representing a preferential loss of intensity of 50% of one of the two alleles in the digested DNA was determined as predominance of one single allele and thus a monoclonal pattern [14]. A discrepancy in XCI pattern represents different clone origins between different tumors while a matching XCI pattern indicates a ½ chance of shared origin.

### Statistical analysis

Statistical analysis was performed using paired *t* test in GraphPad Prisma 5.0.
